# Withholding or continuing glucose-lowering drugs for elective surgery in patients with type 2 diabetes mellitus: a secondary analysis of the MOPED international, prospective, observational study^☆^

**DOI:** 10.1016/j.bja.2026.03.068

**Published:** 2026-05-26

**Authors:** Donal J. Buggy, Malachy O. Columb, Jeroen Hermanides, Markus W. Hollmann, Mark Coburn, Alexander Zarbock, the MOPED Investigators, Angel Caballero, Peter Paal, Sanja Granov, Marijana Rehorić Krkušek, Gianluca Samarani, Tamar Macharadze, Christoph Sponholz, Eleni Arnaoutoglou, Pádraig Ó. Scanaill, Orit Nahtomi Shick, Matteo Bossolasco, Jasmina Ilievska, Jeroen Hermanides, Katarzyna Kotfis, Andreia Capelão, Daniela Ionescu, Sergey Efremov, Pavel Dunts, Unic Stojanovic, Marina Soro, Kemal Tolga Saracoglu, Simon Howell, Paul Köglberger, Gabriel Putzer, Barbara Ebner, Martin Grünbart, Hannes Hoi, Simone Santosuosso, Senada Causevic, Mirza Kovacevic, Asmira Ljuca, Sanja Granov, Ilirijana Haxhibeqiri-Karabdic, Slavenka Straus, Sanja Berić, Ana Jurin Martić, Ana Horvat, Helena Krolo Videka, Emil Dolenc, Jasminka Peršec, Marko Pražetina, Nikola Ivanov, Nataša Paklar, Gianluca Samarani, George Grigolia, Tsotne Samadashvili, Lasha Tabagari, Gvantsa Kachlishvili, Gabriel Janashvili, Johannes Roth, Oliver Sommerfeld, Christoph Sponholz, Lars Bergmann, Sebastian Schmid, Achilles Delis, Marie-Louise Fingerhut, Tobias Hilbert, Gregor Massoth, Claudia Neumann, Maria Wittmann, Christian Koch, Maria Theresa Voelker, Konstantinos Stroumpoulis, Georgia Micha, Georgia Tsaousi, Eleni Arnaoutoglou, Maria Ntalouka, Dimitra Papaspyrou, Styliani Apostolaki, Elissavet Anestiadou, Orestis Ioannidis, Konstantinos Zapsalis, Louise Moran, Ahmed Abdelaatti, Mushtaq Ahmad, Donal Buggy, Pádraig Ó. Scanaill, John Shaker, Junaid Hashmi, Kristiana Nali, Andrea Haren, Sophie Shinnors, Donal Rafferty, Kim Caulfield, Darren McMahon, Yaacov Gozal, Ra'ed Ishaq Jebrin, Waseem Moharib, Orit Nahtomi Shick, Andrea Barranco, Paola Aceto, Ersilia Luca, Andrea Cortegiani, Silvia Fiorelli, Cecilia Menna, Matteo Tiracorrendo, Antonella Cotoia, Annachiara Marra, Valentina Bellini, Elena Bignami, Chiara Domaneschi, Jasmina Ilievska, Abraham H. Hulst, Jeroen Hermanides, Lars IP. Snel, Eelko K. de Groot, Robert Wilpe van, Yara Holtrust, Liedewij Janssen, Emiel Caris, Seppe Koopman, Nieke Oversier, Jonne Postema, Jamila Rapon, Wouter Van Bockel, Samuel Haig Barclay, Marc Bernard Godfried, Tomasz Czarnik, Agata Uchacz, Krzysztof Kusza, Maciej Arciszewski, Katarzyna Kotfis, Maria Pankowiak, Bartosz Pawłowicz, Elżbieta Reszka, Justyna Zajac, Sofia Baptista, Rita Pinto, Susana Andrez, Joana Azevedo, Jose Cardoso, Morais Carina, Rodrigues Elsa, Joana Jones, Fernando José Abelha, Ana Lopes, Joana Mourão, Luís Pereira, Adriana Santos, Florin Teodor Bobirca, Dana Tomescu, Sergiu Sargarovschi, Romina-Marina Sima, Nataliya Efremova, Alexey Ovezov, Ayyaz Hussain, Timur Dzhumatov, Sergey Efremov, Yana Jumatava, Vladimir Skvortsov, Aleksei Trofimov, Dragana Unic-Stojanovic, Marina Soro, José Maria Cubero-Marcos, Gisela Myrella Hermenegildo Chávez, Ignacio Hinojal-Blanco, Ana Martínez-Díaz, Mariona Romero-González, Gerard Urrútia, Ángel Becerra-Bolaños, Héctor Trujillo-Morales, Olga de la Varga-Martínez, Paula Gómez-Aguilar, Ana Nieto-Moreno, Lerzan Dogan, Dilek Ömür Arça, Ayşe Zeynep Turan Civraz, Emine Yurt, Derya Karasu, Tayfun Et, Gaye Boztepe Yilmaz, Osman Ekinci, Deepa Kurup, Sarah Barton, Tim Wilson, Carolina Thomas, James Day, Anil Hormis, Dipesh Patel, Victoria Waugh, Rajesha Srinivasaiah, Nidhi Gautam, Konstantinos Miltsios, Sylvia Daamen, Pierre Harlet, Slama Farsi, Saman Homayun Sepehr, Sophie Debouche, Maxim Van Belle

**Affiliations:** 1 Department of Anesthesiology, Mater Misericordiae University Hospital, School of Medicine, University College Dublin, Ireland; 2 Outcomes Research Consortium, Houston, Texas, USA; 3 Department of Anesthesiology and Intensive Care Medicine, Manchester University Hospitals Foundation NHS Trust, Wythenshawe, UK; 4 Department of Anesthesiology, Research Institutes Amsterdam Cardiovascular Sciences & Amsterdam Public Health, Amsterdam University Medical Centers, Amsterdam, The Netherlands; 5 Department of Anesthesiology and Operative Intensive Care, University Hospital Bonn, Germany; 6 Department of Anesthesiology, Intensive Care and Pain Medicine, University Hospital Münster, Germany; 7 Department of Anesthesiology, University of Texas, McGovern Medical School, Houston, TX, USA

**Keywords:** diabetes mellitus, general anaesthesia, glucose lowering drugs, insulin, postoperative complications, regional anaesthesia

## Abstract

**Background:**

There are conflicting guidelines on whether patients with type 2 diabetes mellitus should withhold or continue glucose-lowering drugs before surgery, especially newer agents. We tested the hypothesis that continuing glucose-lowering drugs increases days alive at home and out of hospital at 30 days (DAH-30).

**Methods:**

We performed a secondary analysis of a prospective, observational study in 21 European countries of patients with type 2 diabetes mellitus undergoing elective surgery. The primary outcome was DAH-30. The exposure of interest was the continuation or discontinuation of glucose-lowering drugs (metformin, sodium-glucose cotransporter 2 inhibitors [SGLT2i], glucagon-like peptide-1 receptor agonists [GLP-1 RA]). Median (interquartile [IQR]) values are shown.

**Results:**

Between January 2021 and December 2024, 5767 participants with type 2 diabetes mellitus (age 64 [range: 22–89] yr; 43% female) were enrolled from 89 centres, with 4988 (87%) undergoing elective surgery. For 3623/5767 (73%) participants receiving metformin, DAH-30 was higher (28 days [24–29]) in 421/3623 (12%) participants who continued metformin on the day of surgery compared with 27 days (23–29) in 3202 of 5767 (88%) participants who had not taken metformin (*P*=0.001). After adjusting for prespecified covariates, continuing metformin on the day of surgery remained associated with higher DAH-30 (0.47 days [95% confidence interval:0.01–0.93]; *P*=0.044). No association was found for either stopping/continuing SGLT2i (*n*=836 participants) or GLP-1 RA (*n*=304 participants) with DAH-30.

**Conclusions:**

Continuing metformin during surgery among patients with type 2 diabetes mellitus was associated with marginally shorter DAH-30, but the sample size for participants receiving SGLT2i and GLP-1 RA therapy precluded any meaningful estimates.

**Tracker ID:**

ESA-IC_CTN_MOPED.

**Editor’s key points**

• There are conflicting guidelines about withholding or continuing non-insulin glucose–lowering drugs before surgery.
• This was a planned secondary analysis of an international, prospective, observational study of the perioperative management of >6000 people with diabetes mellitus across 89 centres in 21 countries.
• Withholding glucose-lowering drugs had no effect on days alive and out of hospital, compared with continuing these drugs.
• These limited observational data suggest patients with type 2 diabetes mellitus should continue to take metformin in the perioperative period.

The global prevalence of diabetes mellitus is increasing, now approaching 10% of the global population, with type 2 diabetes mellitus accounting for >90% of cases.^1^. Complications of type 2 diabetes mellitus occur in >50% patients and frequently require surgical management.^2^^,^^3^ Accordingly, its prevalence is high in the surgical population, varying between 6% and 16% with type of surgery.^4^ Diabetes mellitus is among the most frequent comorbid factors in surgical patients and is associated with increased risk of postoperative complications.^4^^,^^5^ When postoperative complications occur, they are linked with worse long-term outcomes, including increased mortality.^6^

There is an unmet need for evidence on optimal perioperative management of patients with type 2 diabetes mellitus.^7^, ^8^, ^9^ Professional bodies have developed guidelines founded on expert opinion, which are sometimes contradictory.^10^, ^11^ Consequently, there is variability in clinical practice between hospitals and regions.^12^^,^^13^

Non-insulin glucose–lowering drugs (GLD), especially metformin, have been a mainstay of the management of patients with type 2 diabetes mellitus and reduce long-term cardiovascular complications.^14^^,^^15^ New glucose-lowering agents, sodium-glucose co-transporter 2 inhibitors (SGLT2i) and glucagon-like peptide-1 receptor agonists (GLP-1 RA), provide further reduction in long-term complications of patients with type 2 diabetes mellitus.^9^^,^^16^^,^^17^ The reduction of the incidence of heart failure and kidney failure by SGLT2i and the potent weight-reducing abilities of GLP-1 RA have rapidly increased their prescription rates.^18^

However, one of the knowledge deficits in perioperative management of surgical patients with type 2 diabetes mellitus is whether to withhold or continue GLD immediately before surgery. Withholding them could risk deterioration in glycaemic control, with excessively high glucose (>12 mM) potentially facilitating postoperative infectious complications, whereas continuing SGLT2i and GLP-1 RA in fasting people for surgery has been associated with euglycaemic ketoacidosis and delayed gastric emptying, respectively.^19^, ^20^, ^21^ Recent pilot data suggest other potential perioperative benefits of continuing SGLT2i and GLP-1 RA, such as kidney protection and prevention of heart failure.^22^, ^23^, ^24^

MOPED was an international, multicentre, prospective observational study of the management and outcomes of the perioperative journey of people with diabetes across Europe, undergoing a wide range of surgeries, in centres ranging from district general-type hospitals to university teaching and tertiary referral centres.^25^^,^^26^ We undertook this planned, exploratory secondary analysis of the MOPED study to test the hypotheses that patients with type 2 diabetes mellitus whose GLD were continued would have better postoperative outcomes than if they were withheld, because the benefits of these drugs would be maintained.

Our objectives were to test the association between withholding or continuing the three most frequently used GLDs in Europe, metformin, GLP-1 RA and SGLT2i on postoperative 30-day outcomes, with Days at Home at 30 days (DAH-30) as the primary endpoint and postoperative complications (as measured by Clavien-Dindo grade) and dysglycaemia (episodes of glucose >12 or <4 mM) among the secondary outcomes.

## Methods

### Study design

MOPED was a prospective, observational, international, multicentre cohort study. It was registered at ClinicalTrials.gov, number NCT04511312, and documented the perioperative journey of people with diabetes mellitus. Between January 2021 and February 2024, a total of 6196 diabetes mellitus patients in 89 centres across 21 European countries were enrolled. MOPED received institutional review board approval in every country and centre where it was conducted. Specific national and local regulatory authority requirements were followed as applicable.

Participating centres were self-selected by an anaesthesiologist working there. This local investigator enrolled consecutive diabetes mellitus patients presenting for any kind of surgery requiring an anaesthetic technique until their locally determined target number of patients was reached and followed up according to the protocol.^25^^,^^26^

### Inclusion criteria

People with a confirmed diagnosis of type 2 diabetes mellitus, >18 yr, undergoing surgery (defined as requiring any general anaesthesia technique, any specific regional anaesthetic technique or a combination). Ambulatory, elective or emergency surgeries were included. Predefined subgroups of diabetes mellitus patients were highlighted for *a priori* analysis.

### Exclusion criteria

People with gestational diabetes, type 1 diabetes mellitus or any form of diabetes mellitus other than type 2 diabetes mellitus, patients undergoing surgery under local anaesthetic infiltration or topical anaesthesia alone with or without monitored sedation, or surgery not as defined in the inclusion criteria above.

### Primary endpoint

The primary endpoint was DAH-30.^27^^,^^28^

### Secondary endpoints

Secondary endpoints included the comprehensive complications index score based on the Clavien-Dindo scale;^29^ the quality of recovery scale (only taken from patients who are in hospital the day after surgery, i.e. day 1 after surgery);^30^ 30-day mortality; length of hospital stay; length of stay in ICU if applicable; incidence of major adverse events as listed in the European perioperative clinical outcomes definitions manuscript;^31^, ^32^ SORT surgical risk score;^33^ intraoperative and postoperative management of diabetes mellitus; fasting; capillary/arterial blood glucose levels before and < 3 h after surgery; incidence of diabetic ketoacidosis (defined as presence of the triad of glucose >12 mM, ketonaemia >3.0 mM, and lactaemia >2.0 mM); incidence of hypoglycaemia (glucose <4 mM); and duration of use of i.v. insulin infusion therapy.

### Exposure of interest

The exposure of interest was the continuation or discontinuation of glucose-lowering drugs.

### Sample size calculation

The baseline objective was to collect a pragmatic sample of at least 5000 people with diabetes mellitus across at least 50 centres in a minimum of 15 countries. This was based on similar previous projects by the clinical trial network of the *European Society of Anesthesiology & Intensive Care* (ESAIC). It was expected that this should be sufficient for the main epidemiological aspects of this study. From the MOPED study,^26^ there were data on 3623 patients with type 2 diabetes mellitus routinely taking metformin before elective surgery. Metformin was withheld in 3202 (88%) and continued in 421 (12%) patients. We estimated that the study had 88% power to find a 1-day difference in DAH-30, as significant at *P*<0.05 with an empirical standard deviation (SD) of 6.0 as one-fifth of the range.^27^

### Statistical Analyses

Data are presented as mean (SD), median (interquartile) and frequency (%). Results are presented as median or proportion differences with 95% confidence intervals (CI). Continuous data were analysed using Mann–Whitney *U-test,* Hodges–Lehmann and quantile regression statistics for differences in medians, with 95% CI. Categorical data were analysed using differences in proportions with mid *P-*values and 95% CI. Multivariable quantile regression analyses were performed to adjust for relevant patient, diabetic and surgical factors for the effects of GLDs on DAH-30 outcomes.^34^ Data were analysed using Stata 17.0 (StataCorp Inc., College Station, TX, USA) and Number Cruncher Statistical Systems 2024 (NCSS) (NCSS Inc., Kaysville, UT, USA).

## Results

### Participant characteristics

**Fig 1:**
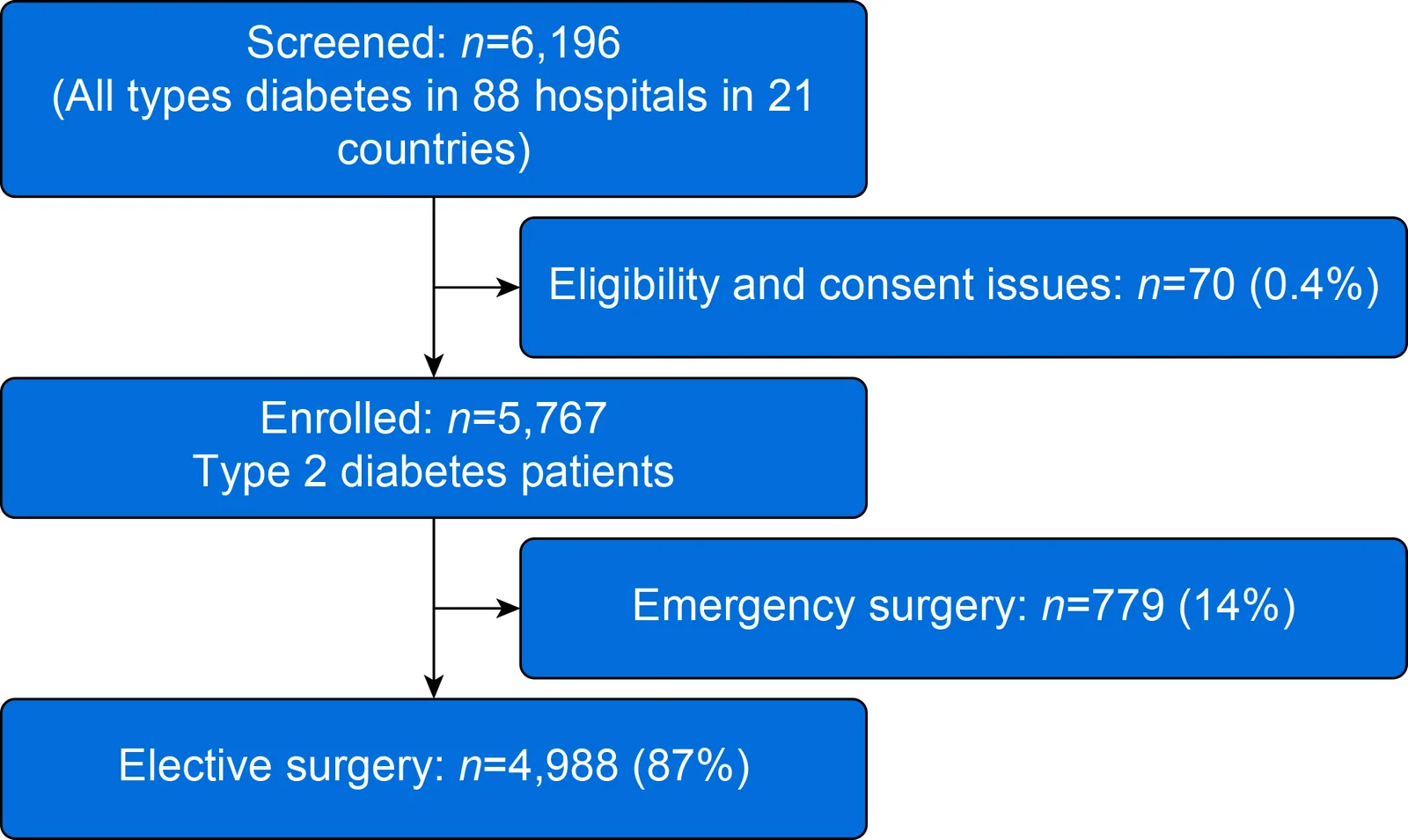
Study flow diagram.

A total of 6196 people were screened from 89 participating centres in 21 countries, of which 6126 were confirmed eligible and enrolled in the study (Fig. 1). Only 12%, 15%, and 14% of participants with type 2 diabetes mellitus continued to take metformin, SGLT2i and GLP-1 RA medications, respectively, on the day of surgery (Table 1). Metformin (73%) was the most common glucose-lowering drug taken, but SGLT2i (17%) and GLP-1 RA (6%) were also taken. We did not document whether participants were on injectable or oral GLP-1 RA.

**Table 1:** Characteristics of type 2 diabetes mellitus cohort undergoing elective surgery. Data are presented as mean (SD), median (interquartiles) and *n* (%).

Variable	Type 2 diabetes mellitus, *n*=4988
**Age (yr)**	63.9 (10.5)
**Height (cm)**	169.1 (9.7)
**Weight (kg)**	84.8 (18.0)
**Body mass index (kg m^−2^)**	29.7 (5.83)
**Sex (male)**	2864 (57.4)
**Ethnicity:**	
**Caucasian**	4434 (89.0)
**African**	66 (1.3)
**Arabic**	68 (1.4)
**Asian**	59 (1.2)
**Other**	358 (7.2)
**American Society of Anesthesiologists physical status (median)**	3.0 (2.0, 3.0)
**American Society of Anesthesiologists physical status**	
**1**	38 (0.8)
**2**	2024 (40.6)
**3**	2702 (54.2)
**4**	210 (4.2)
**5**	13 (0.2)
**Surgical Outcome Risk Tool score**	0.6 (0.2, 1.6)
**Hospital level:**	
**Community**	501 (10.2)
**Private**	277 (5.6)
**Tertiary**	4159 (84.2)
**Duration of diabetes mellitus (yr)**	9.0 (4.0, 16.0)
**Insulin**	1162 (23.3)
**Insulin dose per day (u)**	30.0 (20.0, 50.0)
**Insulin pump *in situ***	9 (0.2)
**Insulin pump 24 h (u)**	45.5 (28.0, 82.8)
**Non-insulin glucose–lowering drugs:**	
**Metformin**	3742 (75)
**Sodium-glucose cotransporter 2 inhibitor**	852 (17)
**Glucagon-like peptide-1 receptor agonist**	301 (6)
**Dipeptidyl peptidase-4 inhibitor**	673 (14)
**α-Glucosidase inhibitor**	15 (0.3)
**Sulphonylurea**	722 (14)
**Other**	131 (3)
**Number per patient**	1.0 (1.0, 2.0)
**Glucose monitoring:**	
**Blood**	2892 (58)
**Urine**	5 (0.1)
**None**	2081 (42)
**Haemoglobin A1c <3 months (mmol mol^−1^)**	50.8 (44.3, 59.6)
**Haemoglobin A1c categories (mmol mol^−1^):**	
**<53**	1311 (26.3)
**53–69**	814 (16.3)
**>69**	311 (6.2)
**Unrecorded**	2552 (51.2)
**Hypoglycaemia events <3 months**	0.0 (0.0, 0.0)
**Ketoacidosis events <3 months**	0.0 (0.0, 0.0)
**Glucose fasting <3 months (mM)**	7.4 (2.47)
**Corticosteroids <6 months**	250 (5.0)
**Corticosteroid drug:**	
**Prednisolone**	167 (68.4)
**Hydrocortisone**	11 (4.5)
**Dexamethasone**	66 (27.1)
**Prednisolone dose (mg)**	90 (5.0, 25.0)
**Prednisolone duration (days)**	60.0 (8.5, 180.0)
**Hydrocortisone dose (mg)**	20.0 (20.0, 150.0
**Hydrocortisone duration (days)**	30.0 (1.5, 30.0)
**Dexamethasone dose (mg)**	8.0 (4.0, 8.0)
**Dexamethasone (days)**	7.0 (3.0, 10.0)
**Creatinine (μM)**	80.0 (66.0, 97.0)

### Unadjusted analyses for prescribed glucose-lowering drugs

Continuing on prescribed metformin was associated with higher DAH-30 (28.0 days [24.0–29.0]), compared with participants in whom these drugs were withheld (27.0 days [23.0, 29.0]); median difference 1.0 (95% CI 0.2, 1.8); *P*=0.01; Table 2). A lower proportion of participants with type 2 diabetes mellitus having ketoacidosis in the first 30 postoperative days was associated with participants continuing metformin (1.4% *vs* 0.2%; difference 1.1% 95% CI 0.0, 1.7; *P*=0.035). The proportion of participants with hypoglycaemia was also associated with continuation of metformin (5.7% *vs* 3.5%; difference 2.4% [95% CI 0.0, 4.0; *P*=0.039]). No episodes of lactic acidosis were recorded. Continuing metformin was also associated with lower peak recorded glucose and the need for supplemental rescue insulin after discharge from postanaesthesia care unit (PACU) (Table 2).

**Table 2:** Metformin status and perioperative 30-day outcomes in type 2 diabetes mellitus and elective surgery. Results are presented as median (interquartiles) and count (%). Data were analysed using Mann–Whitney *U*-test and differences in proportions exact mid *P*-value statistics. DAH-30, days alive at home in first 30 days after surgery; PACU, postanaesthesia care unit.

Endpoints	Withheld (*n*=3202)	Continued (*n*=421)	*P*-value
**DAH-30 (days)**	27.0 (23.0, 29.0)	28.0 (24.0,29.0)	0.0008
**Ketoacidosis**	43/3160 (1.4)	1/415 (0.2)	0.035
**Hypoglycaemia**	181/3159 (5.7)	14/415 (3.4)	0.039
**Clavien-Dindo grade**	*I (0, I)*	*I (0, I)*	0.52
**Diabetes medications increased after surgery**	232/3177 (7.3)	29/417 (7.0)	0.84
**Highest intraoperative glucose (mM)**	8.4 (6.9, 10.5)	8.3 (7.0, 10.4)	0.98
**Lowest intraoperative glucose (mM)**	6.8 (5.6, 8.3)	7.1 (6.0, 8.3)	0.097
**Median glucose in PACU (mM)**	8.0 (6.5, 10.0)	8.2 (6.7, 9.9)	0.77
**Highest glucose post-PACU (mM)**	9.8 (7.9, 12.8)	10.8 (8.4, 13.4)	0.0006
**Lowest glucose post-PACU (mM)**	5.9 (5.0, 7.1)	6.2 (5.2, 7.3)	0.016
**Insulin post-PACU (u)**	0.0 (0.0, 8.3)	0.0 (0.0, 25.0)	0.0004

Continuation or withholding of SGLT2i drugs (Table 3) and GLP-1 RA drugs (Table 4) was not associated with differences in DAH-30. Continuing GLP-1 RA (*n*=46; 15%) was associated with lower blood glucose intraoperatively (*P*=0.039) and post-PACU (*P*=0.0075). Only unadjusted values are provided for dysglycaemia and postoperative complications, given the small cohorts with these findings.

**Table 3:** SGLT2i status and perioperative 30-day outcomes in type 2 diabetes mellitus and elective surgery. Results are presented as median (interquartiles) and count (%). Data were analysed using Mann–Whitney *U*- and differences in proportions exact mid *P*-value statistics. SGLT2i, sodium-glucose cotransporter 2 inhibitor; DAH-30, days alive at home in first 30 days after surgery; PACU, post-anaesthesia care unit.

Endpoints	Withheld (*n*=711)	Continued (*n*=125)	*P*-value
**DAH-30 (days)**	27.0 (23.0, 29.0)	27.0 (24.0, 29.0)	0.71
**Ketoacidosis**	13/699 (1.9)	2/121 (1.6)	1.00
**Hypoglycaemia**	26/701 (3.7)	3/120 (2.4)	0.45
**Clavien-Dindo grade**	*I (0, I)*	*I (I, I)*	0.0002
**Diabetes medications increased after surgery**	53/705 (7.5)	18/124 (14.5)	0.01
**Highest intraoperative glucose (mM)**	8.3 (6.7, 10.2)	8.8 (7.5, 11.3)	0.06
**Lowest intraoperative glucose (mM)**	7.0 (5.8, 8.3)	6.5 (5.4, 7.9)	0.23
**Median glucose in PACU (mM)**	8.0 (6.6, 9.8)	8.3 (6.6, 10.9)	0.23
**Highest glucose post-PACU (mM)**	10.8 (8.4, 13.7)	10.9 (8.3, 14.0)	0.62
**Lowest glucose post-PACU (mM)**	6.1 (5.2, 7.2)	5.5 (4.7, 7.3)	0.045

**Table 4:** GLP-1 RA status and perioperative 30-day outcomes in type 2 diabetes mellitus and elective surgery. Results are presented as median (interquartiles) and count (%). Data were analysed using Mann–Whitney *U*-test and differences in proportions exact mid *P*-value statistics. GLP-1 RA, glucagon-like peptide-1 receptor agonist; DAH-30, days alive at home in first 30 days after surgery; PACU, postanaesthesia care unit.

Endpoints	Withheld (*n*=258)	Continued (*n*=46)	*P*-value
**DAH-30 (days)**	28.0 (23.0, 29.0)	26.0 (24.0, 28.0)	0.18
**Ketoacidosis**	6/244 (2.5)	0/41 (0.0)	0.32
**Hypoglycaemia**	19/243 (7.8)	1/41 (2.4)	0.21
**Clavien-Dindo grade**	*I (0, I)*	*I (I, II)*	0.0032
**Diabetes medications increased after surgery**	21/249 (8.4)	2/41 (4.9)	0.55
**Highest intraoperative glucose (mM)**	8.7 (6.7, 10.8)	7.7 (6.4, 8.5)	0.039
**Lowest intraoperative glucose (mM)**	7.1 (5.8, 8.4)	5.4 (4.9, 6.8)	0.0025
**Median glucose in PACU (mM)**	8.1 (6.4, 10.2)	7.4 (6.8, 9.1)	0.46
**Highest glucose post-PACU (mM)**	10.7 (8.1, 13.8)	8.4 (7.7, 10.1)	0.0075
**Lowest glucose post-PACU (mM)**	6.1 (5.2, 7.4)	5.0 (4.4, 6.4)	0.0052
**Insulin post-PACU (u)**	0.0 (0.0, 30.0)	0.0 (0.0, 10.0)	0.22
**Postoperative pulmonary complications**	7/258 (2.7)	0/46 (0.0)	0.34

### Multivariable analyses

After adjusting for participant characteristics and surgical complexity (Table 5), DAH-30 remained higher for participants who continued metformin (0.47 days [95% CI 0.01, 0.93; *P*=0.044]). Adjusted analyses for SGLT2i and GLP-1 RA drugs did not show any relationship with DAH-30.

**Table 5:** Multivariable analyses of the effect of continuing or withholding metformin, SGLT2i or GLP-1 RA on postoperative outcomes in type 2 diabetes mellitus patients. Data are analysed using multivariable quantile regression with adjusted median differences and 95% confidence intervals (CI). DAH-30, days alive at home in first 30 days after surgery; ASA, American Society of Anesthesiologists; SGLT2, sodium-glucose co-transporter 2; GLP-1 , glucagon-like peptide-1.

DAH-30	Coefficient	95% CI		*P*-value
**Metformin:**				
**Withheld**	Reference			
**Continued**	0.47	0.01	0.93	0.044
**Age (yrs)**	-0.01	-0.03	0.00	0.10
**Sex:**				
**Female**	Reference			
**Male**	0.15	-0.14	0.45	0.31
**Body mass index (kg m-^2^)**	0.06	0.03	0.08	0.000
**Haemoglobin A1c (mmol mol^−1^):**				
**<53**	Reference			
**53–69**	0.02	-0.44	0.47	0.94
**>69**	0.01	-0.66	0.67	0.99
**Unrecorded**	-0.12	-0.46	0.23	0.51
**ASA physical status:**				
1	-2.43	-4.19	-0.66	0.007
**2**	Reference			
3	-0.78	-1.09	-0.47	0.000
4	-4.22	-5.00	-3.43	0.000
5	-3.42	-7.75	0.91	0.12
**Unrecorded**	0.37	-3.17	3.91	0.84
**Surgical complexity:**				
**Minor**	Reference			
**Intermediate**	-0.82	-1.39	-0.24	0.005
**Major**	-1.56	-2.12	-1.01	0.000
**Complex**	-3.54	-4.11	-2.98	0.000
**Hospital recruitment per patient**	0.01	0.00	0.01	0.000
**SGLT2 inhibitor:**				
**Continued**	0.39	-0.42	1.20	0.34
**Age (yr)**	-0.03	-0.06	0.00	0.064
**Sex:**				
**Female**	Reference			
**Male**	0.03	-0.59	0.64	0.94
**Body mass index (kg m^−2^)**	0.04	-0.01	0.09	0.15
**Haemoglobin A1c (mmol mol^−1^):**				
**<53**				
**53–69**	0.66	-0.18	1.50	0.13
**>69**	0.20	-1.00	1.41	0.74
**Unrecorded**	0.23	-0.47	0.94	0.52
**ASA physicalstatus:**				
1	-1.10	-3.92	1.71	0.44
2	Reference			
3	-0.59	-1.23	0.05	0.072
4	-4.01	-5.42	-2.60	0.000
5	-2.48	-10.71	5.75	0.55
**Surgical complexity:**				
**Minor**	Reference			
**Intermediate**	0.01	-1.11	1.12	0.99
**Major**	-0.87	-1.98	0.24	0.13
**Complex**	-2.84	-3.93	-1.75	0.000
**Hospital recruitment per patient**	0.01	0.00	0.01	0.000
**GLP-1 receptor agonist:**				
**Continued**	-0.66	-2.40	1.09	0.46
**Age (yr)**	-0.05	-0.11	0.02	0.16
**Sex:**				
**Female**	Reference			
**Male**	0.05	-1.16	1.25	0.94
**Body mass index (kg m^−2^)**	0.09	0.00	0.18	0.05
**Haemoglobin A1c (mmol mol^−1^):**				
**<53**				
**53–69**	0.22	-1.59	2.03	0.81
**>69**	1.05	-1.40	3.50	0.40
**Unrecorded**	-0.10	-1.61	1.41	0.90
**ASA physical status:**				
**1**	0.92	-5.07	6.91	0.76
**2**	Reference			
**3**	-0.40	-1.74	0.95	0.56
**4**	-8.74	-12.29	-5.20	0.000
**Surgical complexity:**				
**Minor**	Reference			
**Intermediate**	-0.69	-2.94	1.56	0.54
**Major**	-1.75	-4.01	0.50	0.13
**Complex**	-4.41	-6.66	-2.16	0.000
**Hospital recruitment per patient**	0.00	-0.01	0.01	0.62

## Discussion

This secondary analysis of a prespecified *a priori* hypothesis from the MOPED study has shown, in univariate analyses, a significant association between continuing prescribed metformin in the perioperative period and a 1-day improvement in the primary outcome, DAH-30, in patients with type 2 diabetes mellitus undergoing elective surgery. However, after multivariable analysis, adjusting for patient characteristics and surgical complexity, this benefit of DAH-30 in patients continuing metformin was reduced to a 0.5-day (*P*=0.044). There were neutral effects on DAH-30 whether continuing or withholding SGLT2i or GLP-1 RA drugs.

Continuing metformin was associated with less postoperative ketoacidosis and hypoglycaemia. Continuing SGLT2i drugs was associated with increased use of diabetic medications after surgery and an increased Clavien-Dindo grading. Likewise, with continuing GLP-1 RA drugs, there was an increase in Clavien-Dindo grading. On the other hand, continuing GLP-1 RAs was associated with a reduction in intraoperative and postoperative dysglycaemia, compared with withholding them. However, the sizes of the cohorts of patients available for SGLT2i and GLP-1 RA drugs and the event frequencies were too small to draw firm conclusions about dysglycaemia and postoperative complications.

Continuing preoperative metformin was associated with a marginally but significantly improved DAH-30 postoperative outcome. Metformin minimises intraoperative hyperglycaemia^35^ and, in addition to its glucose-lowering effect, metformin has been shown to have other potential cardiovascular health benefits.^36^ There were no reports of metformin-associated lactic acidosis in our data.^37^

However, because this was an observational study, some selection bias could have contributed to these results, considering that people switching from metformin to one of the newer drugs were more likely to have progressive diabetes or heart failure. Taken together, there seems to be a risk-benefit ratio in favour of continuation of metformin use during surgery. However, most patients regularly prescribed metformin stopped taking it on the day of surgery, in contravention of currently available guidelines: Only 14% of patients with type 2 diabetes mellitus prescribed metformin actually took it on the day of surgery, even though the widespread recommendation is that metformin should be continued perioperatively. This highlights a gap between recommendations and implementation, consistent with the variability in practice we observed previously.^26^

Besides their glucose-lowering effects, SGLT2i have been associated with beneficial effects on heart failure and renal failure.^24^^,^^38^^,^^39^ In addition, ceasing SGLT2i in people with heart failure may be associated with rebound heart failure.^40^ We observed that continuing SGLT2i marginally increased postoperative complications at 30 days, but not as a consequence of euglycaemic ketoacidosis, of which we had zero cases. Again, selection bias should be considered, since patients continuing SGLT2i may have been among those with heart failure. Unfortunately, we could not distinguish between people receiving SGLT2i for diabetes, heart failure or both. Thus, our data should not change current messaging to adhere to existing recommendations, therefore withholding SGLT2i for 48 hours prior to surgery if prescribed for diabetes mellitus, and continuing them perioperatively for those being treated for heart failure in the absence of diabetes mellitus.

Finally, although continuing GLP-1 RA in the perioperative period had an anticipated glucose-lowering effect,^41^ the cohort and number of events were low. These observations are unlikely to be attributable to a selection bias effect, because there is no clear incentive to stop GLP-1 RA apart from aspiration risk. Among people with diabetes mellitus, both SGLT2i and GLP-1RA have been shown to positively affect long-term cardiovascular and renal outcomes among patients with type 2 diabetes mellitus.^38^^,^^39^ Regarding aspiration, there was no reported case among our patients, whether taking GLP-1 RA or not. There were no additional postoperative pulmonary complications among people continuing them, consistent with recent retrospective cohort studies.^42^

This study is limited by the fact that a relatively small number of self-selecting hospitals, relative to the entire health service of that country, may not have been truly representative of their countries’ overall national practice of managing diabetes mellitus perioperatively. The time window for consecutive individuals with diabetes mellitus inclusion (typically 6–9 months) was selected locally by collaborators in participating hospitals and occurred over a 3-year period, delayed by the COVID-19 pandemic, rather than in a defined epoch for all hospitals simultaneously. Therefore, the data could have been affected by the inexorable progression in practice changes in surgery or endocrinology, which could have occurred variably in different countries. It is also interesting to note that there were significant positive associations between increasing DAH-30 and patient enrolment by centres, which perhaps suggests that improved outcomes are associated with increased workload and expertise in centres.

Although the collection of 30 day outcome data was at least partly supported by hospital documentation, in most cases it was dependent on patients with diabetes mellitus self-reporting their 30 day health experience to clinician researchers through telephone calls. Although we did not document race, our study population is likely to be predominantly White Caucasian, and therefore our findings may not be entirely applicable to other racial types. In addition, our primary outcome measure, DAH-30, a validated patient-centric measure of postoperative outcome, has been shown to have a minimal clinically meaningful difference of 3 days.^43^ Although this suggests that our finding of a 1-day, or even a 0.5-day, median difference in DAH-30 is not clinically important, nonetheless, from the patient perspective, any increased hospital bed occupancy represents an increase in healthcare costs. Also, for the purposes of this study, small differences are of interest from an estimation or measurement perspective. Moreover, this is the first large-scale study to prospectively evaluate associations between continuing or withholding the three most prominent GLDs and immediate and 30-day postoperative outcomes and provides reassuring data for adhering to existing guidelines on perioperative management of patients with diabetes mellitus who are on GLDs.

In summary, although almost 75% of patients with type 2 diabetes mellitus undergoing surgery in Europe continue to be on metformin, the number on other GLDs, such as SGLT2i or GLP-1 RA, is approaching 20%. Continuing metformin for elective surgery with type 2 diabetes mellitus is not associated with adverse outcomes, but may be beneficial. Withholding or continuing SGLT2i and GLP-1 RA perioperatively had a neutral effect on DAH-30, but the cohorts were too small to interpret effects on dysglycaemia and postoperative complications.

## Authors' contributions

Conceptualisation, study design, peer-reviewed application to ESA-IC securing sponsorship, further CAI-BJA funding, literature search, creation of e-CRF, data collection, data curation, methodology, directly accessed and verified the underlying data reported, data interpretation, project administration, resources, supervision, validation, writing – original draft, writing–review & editing: DJB.

Study design input, e-CRF, data curation, methodology, statistical analysis, software, directly accessed and verified the underlying data reported, writing–review & editing: MOC

Study design input, literature search, e-CRF, data collection, methodology, supervision, writing–review & editing: JH

Study design input, creation of e-CRF, data collection, methodology, supervision, writing–review & editing: MWH

Methodology, supervision, writing–review & editing: MC

Study design input, literature search, data collection, methodology, supervision, writing–review & editing: AZ

Full access to the raw data: all authors

Accessed and verified the database: MC, DJB

The complete list of collaborating investigators is listed in the Supplementary Material

## Copyright

Copyright rests with ESA-IC and the steering-writing committee.

## Transparency statement

DJB, the lead author and manuscript’s guarantor, affirms that this manuscript is an honest, accurate, and transparent account of the MOPED study being reported. It is the first and most important study from the MOPED study database, reporting the primary outcomes and main *a priori* hypotheses. It is anticipated that further manuscripts will be generated from secondary analyses in due course.

## Funding

MOPED was predominantly sponsored by a grant from the European Society of Anaesthesiology and Intensive Care (ESA-IC) Clinical Trial Network (Track Number ESA-IC_CTN_MOPED). The ESAIC CTN provides an infrastructure for clinical research in the fields of Anaesthesia, Pain and Intensive Care by transnational European collaborative studies.

The ESA-IC had no role in the overall study design, data analysis, data interpretation, or writing of this manuscript. Enrolment in Ireland and the UK was partially funded by a joint grant (€100,000) from College of Anaesthesiologists of Ireland (CAI) and British Journal of Anaesthesia (BJA).

## Declarations of Interest

All authors have completed the ICMJE uniform disclosure form at www.icmje.org/ coi_disclosure.pdf. Authors’ financial or other relationships with independent organisations in the previous 3 years are shown in the ICMJE forms and may be summarised as follows: DJB is an editorial board member of the British Journal of Anaesthesia (BJA) and honorary president of College of Anaesthesiologists of Ireland. MOC received ESA-IC grant to fund statistical software and received honoraria as editorial board member of the European Journal of Anaesthesiology and as chair of the research methods masterclass of the ESA-IC. JH received grants from ESA-IC and ZonMw. MWH received grants from ESA-IC and ZonMw and is a director of IARS and section editor in journals Anesthesia & Analgesia and Frontiers in Physiology. MC received honoraria for lectures by Fresenius and Baxter and DFG. AZ received free biomarker support and research grant from Biomerieux. Additional research grants were obtained from Baxter. AZ has also received consultancy fees from Alexia, AM Pharma, Paion, Novartis, Guard Therapeutics, Baxter, Bayer, Biomerieux, Fresenius, and Viatris.

## List of National Coordinators

**Andorra** – Angel Caballero

**Austria** – Peter Paal

**Bosnia and Herzegovina** – Sanja Granov

**Croatia** – Marijana Rehorić Krkušek

**France** – Gianluca Samarani

**Georgia** – Tamar Macharadze

**Germany** – Christoph Sponholz

**Greece** – Eleni Arnaoutoglou

**Ireland** – Pádraig Ó Scanaill

**Israel** – Orit Nahtomi Shick

**Italy** – Matteo Bossolasco

**Macedonia** – Jasmina Ilievska

**Netherlands** – Jeroen Hermanides

**Poland** – Katarzyna Kotfis

**Portugal** – Andreia Capelão

**Romania** – Daniela Ionescu

**Russia** – Sergey Efremov

**Russia** – Pavel Dunts

**Serbia**– Unic Stojanovic

**Spain** – Marina Soro

**Turkey** – Kemal Tolga Saracoglu

**United Kingdom** – Simon Howell

**Collaborator list: MOPED**

Andorra:

**Hospital Nostra Senyora de Meritxell**: Angel Caballero

Austria:

**Klinikum Wels-Grieskirchen GmbH**: Paul Köglberger
**Medical University Innsbruck**: Gabriel Putzer
**St. John of God Hospital, Paracelsus Medical University**: Barbara Ebner, Martin Grünbart, Hannes Hoi
**Universitätsklinikum Sankt Pölten**: Simone Santosuosso

Bosnia and Herzegovina:

**Cantonal Hospital Zenica**: Senada Causevic, Mirza Kovacevic, Asmira Ljuca
**Clinical Center University of Sarajevo**: Sanja Granov, Ilirijana Haxhibeqiri-Karabdic, Slavenka Straus

Croatia:

**Clinical hospital Sveti Duh**: Sanja Berić
**County hospital Čakovec**: Ana Jurin Martić
**UH Sestre milosrdnice**: Ana Horvat, Helena Krolo Videka
**University hospital Dubrava**: Emil Dolenc, Jasminka Peršec, Marko Pražetina
**University Hospital Merkur**: Nikola Ivanov, Nataša Paklar, Marijana Rehorić Krkušek

France:

**Hopital des Pays du Mont Blanc**: Gianluca Samarani

Georgia:

**5th Clinical Hospital Open Heart**: George Grigolia
**Caucasus Medical Centre**: Tsotne Samadashvili
**Chapidze Emergency Cardiology Center**: Lasha Tabagari
**MediClubGeorgia**: Gvantsa Kachlishvili
**Oftalmiji**: Gabriel Janashvili

Germany:

**Jena University Hospital**: Johannes Roth, Oliver Sommerfeld, Christoph Sponholz
**Universitätsklinikum Knappschaftskrankenhaus Bochum**: Lars Bergmann
**University Hospital Ulm**: Sebastian Schmid
**University Hospital Bonn**: Achilles Delis, Marie-Louise Fingerhut, Tobias Hilbert, Gregor Massoth, Claudia Neumann, Maria Wittmann
**University Hospital Giessen**: Christian Koch
**University of Leipzig Medical Center**: Maria Theresa Voelker

Greece:

**"Helena Venizelou” General and Maternity Hospital of Athens, Greece**: Konstantinos Stroumpoulis, Georgia Micha
**AHEPA University Hospital**: Georgia Tsaousi
**Faculty of Medicine, University of Thessaly, Larissa University Hospital**: Eleni Arnaoutoglou, Maria Ntalouka, Dimitra Papaspyrou
**General Hospital of Chania**: Styliani Apostolaki
**General Hospital of Thessaloniki "George Papanikolaou"**: Elissavet Anestiadou, Orestis Ioannidis, Konstantinos Zapsalis

Ireland:

**Letterkenny University Hospital**: Louise Moran
**Mater Misericordiae University Hospital**: Ahmed Abdelaatti, Mushtaq Ahmad, Donal Buggy, Pádraig Ó Scanaill, John Shaker
**Our Lady of Lourdes Hospital**: Junaid Hashmi
**South Infirmary Victoria University Hospital (SIVUH)**: Kristiana Nali
**St Vincent's University Hospital**: Andrea Haren
**Tallaght University Hospital**: Sophie Shinnors
**University College Hospital Galway**: Donal Rafferty
**University Hospital Waterford**: Kim Caulfield, Darren McMahon

Israel:

**Shaare Zedek Medical Center**: Yaacov Gozal, Ra'ed Ishaq Jebrin, Waseem Moharib, Orit Nahtomi Shick

Italy:

**A.R.N.A.S Civico**: Andrea Barranco
**Fondazione Policlinico Agostino Gemelli IRCCS**: Paola Aceto, Ersilia Luca
**Policlinico Paolo Giaccone. University of Palermo**: Andrea Cortegiani
**Sant’Andrea Hospital - Sapienza University of Rome**: Silvia Fiorelli, Cecilia Menna, Matteo Tiracorrendo
**University of Foggia**: Antonella Cotoia
**University of naples federico II**: Annachiara Marra
**University of Parma**: Valentina Bellini, Elena Bignami, Chiara Domaneschi

Macedonia:

**University Clinic State Cardiosurgery**: Jasmina Ilievska

Netherlands:

**Amsterdam UMC location AMC**: Abraham H Hulst, Jeroen Hermanides, Lars IP Snel, Eelko K de Groot, Robert Wilpe van
**Amsterdam UMC, location VUmc**: Yara Holtrust, Liedewij Janssen
**Maasstad Hospital**: Emiel Caris, Seppe Koopman, Nieke Oversier, Jonne Postema, Jamila Rapon, Wouter Van Bockel
**OLVG**: Samuel Haig Barclay, Marc Bernard Godfried

Poland:

**Department of Anesthesiology and Intensive Care, Institute of Medical Sciences, University of Opole**: Tomasz Czarnik, Agata Uchacz
**Hospital of Unversity Medical Sciences Poznan**: Krzysztof Kusza
**Pomeranian Medical University**: Maciej Arciszewski, Katarzyna Kotfis, Maria Pankowiak, Bartosz Pawłowicz, Elżbieta Reszka
**Regional Specialist Hospital in Wroclaw**: Justyna Zajac

Portugal:

**Centro Hospitalar de Setúbal, EPE**: Andreia Capelão
**Hospital Beatriz Ângelo**: Sofia Baptista, Rita Pinto
**Hospital da Luz - Setubal**: Susana Andrez, Joana Azevedo, Jose Cardoso, Morais Carina, Rodrigues Elsa, Joana Jones
**HOSPITAL DE SÃO JOÃO**: Fernando José Abelha, Ana Lopes, Joana Mourão, Luís Pereira
**Unidade Local De Saúde Do Alentejo Central**: Adriana Santos

Romania:

**Dr Ion Cntacuzino Clinical Hospital**: Florin Teodor Bobirca
**Fundeni Clinical Institute**: Dana Tomescu
**Institutul Regional de Gastroenterologie şi Hepatologie, Cluj Napoca:** Sergiu Sargarovschi
**St John Hospital, Bucur Maternity**: Romina-Marina Sima

Russian:

**Moscow Regional Research Clinical Institute**: Nataliya Efremova, Alexey Ovezov
**Northern State Medical University and City hospital 1 named after E.E. Volosevich**: Ayyaz Hussain
**Saint-Petersburg State University Hospital**: Timur Dzhumatov, Sergey Efremov, Yana Jumatava, Vladimir Skvortsov, Aleksei Trofimov

Serbia:

**Cardiovascular Institute Dedinje**: Dragana Unic-Stojanovic

Spain:

**Hospital Clinico Universitario De Valencia**: Marina Soro
**Hospital de la Santa Creu i Sant Pau**: José Maria Cubero-Marcos, Gisela Myrella Hermenegildo Chávez, Ignacio Hinojal-Blanco, Ana Martínez-Díaz, Mariona Romero-González, Gerard Urrútia
**Hospital Universitario de Gran canaria doctor negrin**: Ángel Becerra-Bolaños, Héctor Trujillo-Morales
**Hospital Universitario Infanta Leonor**: Olga de la Varga-Martínez, Paula Gómez-Aguilar, Ana Nieto-Moreno

Turkey:

**Acibadem Altunizade Hospital**: Lerzan Dogan
**Dokuz Eylul University**: Dilek Ömür Arça
**Health And Science University Derince Training And Research Hospital**: Ayşe Zeynep Turan Civraz, Emine Yurt
**Health Sciences University Bursa Yuksek Ihtisas Training and Research Hospital**: Derya Karasu
**Karaman Training and Research Hospital**: Tayfun Et
**Kartal Dr. Lutfi Kirdar Training and Research Hospital**: Gaye Boztepe Yilmaz, Kemal Tolga Saracoglu
**University of Health Sciences Turkey**: Osman Ekinci

United Kingdom:

**Barnsley Hospital NHSFT**: Deepa Kurup
**Basildon and Thurrock University Hospitals**: Sarah Barton
**Doncaster and Bassetlaw Teaching Hospitals NHS Foundation Trust**: Tim Wilson
**Leeds Teaching Hospitals NHS Trust**: Carolina Thomas
**Oxford University Hospitals**: James Day
**Rotherham Hospital**: Anil Hormis
**Royal Free London NHS Foundation Trust**: Dipesh Patel
**Royal Liverpool site of Liverpool University Hospitals Foundation Trust**: Victoria Waugh
**The Walton Centre NHS Foundation Trust**: Rajesha Srinivasaiah
**West Hertfordshire Teaching Hospitals**: Nidhi Gautam
**Whittington Hospital**: Konstantinos Miltsios

**Management Team**

**European Society of Anaesthesiology and Intensive Care, Brussels:**

Sylvia Daamen, Pierre Harlet, Slama Farsi, Saman Homayun Sepehr, Sophie Debouche and Maxim Van Belle
