# Supplementary material for: Withholding or continuing glucose-lowering drugs for elective surgery in patients with type 2 diabetes mellitus: a secondary analysis of the MOPED international, prospective, observational study
Source: Br J Anaesth. 2026 May 26;137(1):99–109. doi: 10.1016/j.bja.2026.03.068 (PMC13494077; doi:10.1016/j.bja.2026.03.068)
Supplement: Supplementary file 1 [file mmc1.docx]

# List of National Coordinators

**Andorra** –Angel Caballero

**Austria** –Peter Paal

**Bosnia and Herzegovina** - Sanja Granov

**Croatia** – Marijana Rehorić Krkušek

**France**	- Gianluca Samarani

**Georgia** - Tamar Macharadze

**Germany** - Christoph Sponholz

**Greece** – Eleni Arnaoutoglou

**Ireland** - Pádraig Ó Scanaill

**Israel** - Orit Nahtomi Shick

**Italy** - Matteo Bossolasco

**Macedonia** –Jasmina Ilievska

**Netherlands** –Jeroen Hermanides

**Poland** –Katarzyna Kotfis

**Portugal** - Andreia Capelão

**Romania** –Daniela Ionescu

**Russia** –Sergey Efremov

**Russia** –Pavel Dunts

**Serbia**	- Unic Stojanovic

**Spain** –Marina Soro

**Turkey** –Kemal Tolga Saracoglu

**United Kingdom** –Simon Howell

**Collaborator list: MOPED**

Andorra:

- **Hospital Nostra Senyora de Meritxell**: Angel Caballero

Austria:

- **Klinikum Wels-Grieskirchen GmbH**: Paul Köglberger
- **Medical University Innsbruck**: Gabriel Putzer
- **St. John of God Hospital, Paracelsus Medical University**: Barbara Ebner, Martin Grünbart, Hannes Hoi
- **Universitätsklinikum Sankt Pölten**: Simone Santosuosso

Bosnia and Herzegovina:

- **Cantonal Hospital Zenica**: Senada Causevic, Mirza Kovacevic, Asmira Ljuca
- **Clinical Center University of Sarajevo**: Sanja Granov, Ilirijana Haxhibeqiri-Karabdic, Slavenka Straus

Croatia:

- **Clinical hospital Sveti Duh**: Sanja Berić
- **County hospital Čakovec**: Ana Jurin Martić
- **UH Sestre milosrdnice**: Ana Horvat, Helena Krolo Videka
- **University hospital Dubrava**: Emil Dolenc, Jasminka **Peršec**, Marko Pražetina
- **University Hospital Merkur**: Nikola Ivanov, Nataša Paklar, Marijana Rehorić Krkušek

France:

- **Hopital des Pays du Mont Blanc**: Gianluca Samarani

Georgia:

- **5th Clinical Hospital Open Heart**: George Grigolia
- **Caucasus Medical Centre**: Tsotne Samadashvili
- **Chapidze Emergency Cardiology Center**: Lasha Tabagari
- **MediClubGeorgia**: Gvantsa Kachlishvili
- **Oftalmiji**: Gabriel Janashvili

Germany:

- **Jena University Hospital**: Johannes Roth, Oliver Sommerfeld, Christoph Sponholz
- **Universitätsklinikum Knappschaftskrankenhaus Bochum**: Lars Bergmann
- **University Hospital Ulm**: Sebastian Schmid
- **University Hospital Bonn**: Achilles Delis, Marie-Louise Fingerhut, Tobias Hilbert, Gregor Massoth, Claudia Neumann, Maria Wittmann
- **University Hospital Giessen**: Christian Koch
- **University of Leipzig Medical Center**: Maria Theresa Voelker

Greece:

- **"Helena Venizelou" General and Maternity Hospital of Athens, Greece**: Konstantinos Stroumpoulis, Georgia Micha
- **AHEPA University Hospital**: Georgia Tsaousi
- **Faculty of Medicine, University of Thessaly, Larissa University Hospital**: Eleni Arnaoutoglou, Maria Ntalouka, Dimitra Papaspyrou
- **General Hospital of Chania**: Styliani Apostolaki
- **General Hospital of Thessaloniki "George Papanikolaou"**: Elissavet Anestiadou, Orestis Ioannidis, Konstantinos Zapsalis

Ireland:

- **Letterkenny University Hospital**: Louise Moran
- **Mater Misericordiae University Hospital**: Ahmed Abdelaatti, Mushtaq Ahmad, Donal Buggy, Pádraig Ó Scanaill, John Shaker
- **Our Lady of Lourdes Hospital**: Junaid Hashmi
- **South Infirmary Victoria University Hospital (SIVUH)**: Kristiana Nali
- **St Vincent's University Hospital**: Andrea Haren
- **Tallaght University Hospital**: Sophie Shinnors
- **University College Hospital Galway**: Donal Rafferty
- **University Hospital Waterford**: Kim Caulfield, Darren McMahon

Israel:

- **Shaare Zedek Medical Center**: Yaacov Gozal, Ra'ed Ishaq Jebrin, Waseem Moharib, Orit Nahtomi Shick

Italy:

- **A.R.N.A.S Civico**: Andrea Barranco
- **Fondazione Policlinico Agostino Gemelli IRCCS**: Paola Aceto, Ersilia Luca
- **Policlinico Paolo Giaccone. University of Palermo**: Andrea Cortegiani
- **Sant’Andrea Hospital - Sapienza University of Rome**: Silvia Fiorelli, Cecilia Menna , Matteo Tiracorrendo
- **University of Foggia**: Antonella Cotoia
- **university of naples federico II**: Annachiara Marra
- **University of Parma**: Valentina Bellini, Elena Bignami, Chiara Domaneschi

Macedonia:

- **University Clinic State Cardiosurgery**: Jasmina Ilievska

Netherlands:

- **Amsterdam UMC location AMC**: Abraham H Hulst, Jeroen Hermanides, Lars IP Snel, Eelko K de Groot, Robert Wilpe van
- **Amsterdam UMC, location VUmc**: Yara Holtrust, Liedewij Janssen
- **Maasstad Hospital**: Emiel Caris, Seppe Koopman, Nieke Oversier, Jonne Postema, Jamila Rapon, Wouter Van Bockel
- **OLVG**: Samuel Haig Barclay, Marc Bernard Godfried

Poland:

- **Department of Anesthesiology and Intensive Care, Institute of Medical Sciences, University of Opole**: Tomasz Czarnik, Agata Uchacz
- **Hospital of Unversity Medical Sciences Poznan**: Krzysztof Kusza
- **Pomeranian Medical University**: Maciej Arciszewski, Katarzyna Kotfis , Maria Pankowiak, Bartosz Pawłowicz , Elżbieta Reszka
- **Regional Specialist Hospital in Wroclaw**: Justyna Zajac

Portugal:

- **Centro Hospitalar de Setúbal, EPE**: Andreia Capelão
- **Hospital Beatriz Ângelo**: Sofia Baptista, Rita Pinto
- **Hospital da Luz - Setubal**: Susana Andrez, Joana Azevedo, Jose Cardoso, Morais Carina, Rodrigues Elsa, Joana Jones
- **HOSPITAL DE SÃO JOÃO**: Fernando José Abelha, Ana Lopes, Joana Mourão, Luís Pereira
- **Unidade Local De Saúde Do Alentejo Central**: Adriana Santos

Romania:

- **Dr Ion Cntacuzino Clinical Hospital**: Florin Teodor Bobirca
- **Fundeni Clinical Institute**: Dana Tomescu
- **Institutul Regional de Gastroenterologie și Hepatologie, Cluj Napoca:** Sergiu Sargarovschi
- **St John Hospital, Bucur Maternity**: Romina-Marina Sima

Russian:

- **Moscow Regional Research Clinical Institute**: Nataliya Efremova, Alexey Ovezov
- **Northern State Medical University and City hospital 1 named after E.E. Volosevich**: Ayyaz Hussain
- **Saint-Petersburg State University Hospital**: Timur Dzhumatov , Sergey Efremov, Yana Jumatava, Vladimir Skvortsov, Aleksei Trofimov

Serbia:

- **Cardiovascular Institute Dedinje**: Dragana Unic-Stojanovic

Spain:

- **Hospital Clinico Universitario De Valencia**: Marina Soro
- **Hospital de la Santa Creu i Sant Pau**: José Maria Cubero-Marcos, Gisela Myrella Hermenegildo Chávez, Ignacio Hinojal-Blanco , Ana Martínez-Díaz , Mariona Romero-González, Gerard Urrútia
- **Hospital Universitario de Gran canaria doctor negrin**: Ángel Becerra-Bolaños, Héctor Trujillo-Morales
- **Hospital Universitario Infanta Leonor**: Olga de la Varga-Martínez, Paula Gómez-Aguilar, Ana Nieto-Moreno

Turkey:

- **Acibadem Altunizade Hospital**: Lerzan Dogan
- **Dokuz Eylul University**: Dilek Ömür Arça
- **Health And Science University Derince Training And Research Hospital**: Ayşe Zeynep Turan Civraz, Emine Yurt
- **Health Sciences University Bursa Yuksek Ihtisas Training and Research Hospital**: Derya Karasu
- **Karaman Training and Research Hospital**: Tayfun Et
- **Kartal Dr. Lutfi Kirdar Training and Research Hospital**: Gaye Boztepe Yilmaz, Kemal Tolga Saracoglu
- **University of Health Sciences Turkey**: Osman Ekinci

United Kingdom:

- **Barnsley Hospital NHSFT**: Deepa Kurup
- **Basildon and Thurrock University Hospitals**: Sarah Barton
- **Doncaster and Bassetlaw Teaching Hospitals NHS Foundation Trust**: Tim Wilson
- **Leeds Teaching Hospitals NHS Trust**: Carolina Thomas
- **Oxford University Hospitals**: James Day
- **Rotherham Hospital**: Anil Hormis
- **Royal Free London NHS Foundation Trust**: Dipesh Patel
- **Royal Liverpool site of Liverpool University Hospitals Foundation Trust**: Victoria Waugh
- **The Walton Centre NHS Foundation Trust**: Rajesha Srinivasaiah
- **West Hertfordshire Teaching Hospitals**: Nidhi Gautam
- **Whittington Hospital**: Konstantinos Miltsios

**Management Team**

**European Society of Anaesthesiology and Intensive Care, Brussels:**

Sylvia Daamen, Pierre Harlet, Slama Farsi, Saman Homayun Sepehr, Sophie Debouche and Maxim Van Belle
